# IoT-Based Airport Noise Perception and Monitoring: Multi-Source Data Fusion, Spatial Distribution Modeling, and Analysis

**DOI:** 10.3390/s25082347

**Published:** 2025-04-08

**Authors:** Jie Liu, Shiman Sun, Ke Tang, Xinyu Fan, Jihong Lv, Yinxiang Fu, Xinpu Feng, Liang Zeng

**Affiliations:** 1 Chongqing Airport Group Co., Ltd., Chongqing 401120, China; 15310850011@163.com (J.L.); fanxinyu199911@outlook.com (X.F.); 18983287870@163.com (Y.F.); 2 Civil Aviation Research Base (Beijing) Co., Ltd., Beijing 100621, China; jxcrssm@163.com (S.S.); mhkyjd@163.com (J.L.); fengxinpu@163.com (X.F.); 3 School of Air Traffic Management, Civil Aviation Flight University of China, Guanghan 618307, China; allen18708422558@163.com

**Keywords:** Internet of Things (IoT), multi-source data fusion, spatial distribution modeling, airport noise monitoring, kriging interpolation

## Abstract

With the acceleration of global urbanization, airport noise pollution has emerged as a significant environmental concern that demands attention. Traditional airport noise monitoring systems are fraught with limitations, including restricted spatial coverage, inadequate real-time data acquisition capabilities, poor data correlation, and suboptimal cost-effectiveness. To address these challenges, this paper proposes an innovative airport noise perception and monitoring approach leveraging Internet of Things (IoT) technology. This method integrates multiple data streams, encompassing noise, meteorological, and ADS–B data, to achieve precise noise event tracing and deep multi-source data fusion. Furthermore, this study employs Kriging interpolation and Inverse Distance Weighting (IDW) techniques to perform spatial interpolation on data from sparse monitoring sites, thereby constructing a spatial distribution model of airport noise. The results of the practical application demonstrate that the proposed airport noise monitoring method can accurately reflect the spatiotemporal distribution patterns of airport noise and effectively correlate noise events, thereby providing robust data support for the development of airport noise control policies.

## 1. Introduction

The escalating volume of air traffic has thrust airport noise pollution into the spotlight as a salient environmental issue in the trajectory of global urbanization. Empirical evidence has illuminated a substantial augmentation in noise exposure within communities encircling airports. Aircraft noise, with its diverse intensities, inflicts deleterious effects on the human nervous system and mental health [1], while also profoundly compromising residents’ sleep quality, cardiovascular health, and the ecological milieu [2,3]. The World Health Organization (WHO) has underscored that environmental noise exposure can detrimentally affect residents’ physical and mental health, precipitating a spectrum of issues, including sleep disorders, psychological distress, cognitive deficits, hearing impairment, and tinnitus [4,5]. In the purview of airport noise research, a case in point is Congonhas Airport in Brazil, where background noise levels surpass regulatory benchmarks even during its closed hours (after 23:00), with certain periods witnessing noise levels that exceed permissible limits by six dB(A) and diverge significantly from ambient noise levels [6]. Additionally, field surveys conducted around Frankfurt Airport have unveiled a pronounced negative correlation between aircraft noise and community social quality of life, encompassing aspects such as satisfaction with infrastructure and regional attractiveness, with airport expansion further exacerbating noise disturbances for residents [7,8]. As a principal source of noise, airports exert a broad impact range and enduring duration. Against the backdrop of relentless growth in air transport demand, this issue is becoming increasingly pronounced. Airport noise also adversely affects the ecological environment by disrupting wildlife habitats and migration patterns. Moreover, it can impede economic development in airport vicinities, restrict land use planning, and amplify social conflicts. Thus, airport noise monitoring emerges as a pivotal task for environmental stewardship and an urgent imperative to safeguard public health.

The International Civil Aviation Organization (ICAO) and environmental agencies worldwide have underscored the significance of airport noise management, advocating for the deployment of scientific monitoring and modeling techniques to accurately quantify noise exposure levels and to formulate effective mitigation strategies based on robust data. Traditional airport noise monitoring systems, which predominantly utilize fixed-location sound level meters, are hampered by substantial limitations in spatial coverage, real-time data acquisition, and cost-effectiveness. For instance, fixed monitoring sites are incapable of comprehensively capturing the intricate spatial distribution characteristics of noise, particularly under complex topographical or meteorological conditions where noise propagation patterns can undergo substantial alterations. Furthermore, existing methodologies often rely exclusively on single noise data sources while overlooking the pivotal influence of meteorological conditions (such as wind speed, temperature, and humidity) and flight dynamics (such as flight altitude, speed, and route) on noise distribution, thereby compromising the accuracy and practical applicability of the monitoring outcomes. Despite these challenges, traditional sound level meters remain incapable of achieving multi-point real-time monitoring. The mechanisms by which meteorological conditions (such as wind speed and temperature gradients) modulate noise propagation have yet to be fully elucidated. Additionally, there exists a notable deficiency in the optimization of monitoring site layouts and the precision of noise mapping [9].

The evolution of airport noise monitoring technology has traversed an extensive period of exploration and development. In its nascent stages, the field predominantly relied on rudimentary manual monitoring methods and basic sound level meters. These early techniques, such as traditional sound level technology and rudimentary noise mapping, were employed for airport noise assessment [10]. However, with the advent of continuous technological advancements, automated monitoring systems have progressively emerged as the predominant technology within this domain. Notable examples include O’Hare International Airport in Chicago, IL, USA, and Heathrow Airport in London, UK, both of which have established relatively comprehensive automated noise monitoring systems. These advanced systems are capable of continuous noise level measurements around airports and can conduct integrated analyses with meteorological data and flight information. In China, major airports such as Beijing Capital International Airport, Guangzhou Baiyun International Airport, and Shanghai Hongqiao International Airport have also constructed automated noise monitoring systems. While these systems can provide a certain level of noise monitoring data, they still exhibit limitations in data processing, multi-source data fusion, and intelligent analysis.

Traditional noise monitoring methods, constrained by low equipment deployment density and insufficient data correlation, are incapable of achieving fine-grained identification of noise events and precise prediction of their spatial distribution. In recent years, the rapid advancement of Internet of Things (IoT) technology has paved a novel research pathway for high-resolution noise perception. Through the collaborative operation of multimodal sensors and the application of data fusion techniques, the dynamics and reliability of noise monitoring can be significantly enhanced [11,12]. IoT technology facilitates automatic data collection and transmission via sensor networks, thereby greatly improving the efficiency and accuracy of noise monitoring. Wireless Sensor Networks (WSN), widely used in environmental monitoring, can be deployed around airports to achieve real-time noise data collection and transmission through ARM control modules [13,14,15]. For example, the ANOMS system at Warsaw Chopin Airport integrates 10 distributed monitoring sites, combining meteorological sensors and ADS–B (Automatic Dependent Surveillance–Broadcast) data to accurately correlate noise events with specific flights [16]. The precise identification of noise events necessitates the integration of acoustic, ADS–B (Automatic Dependent Surveillance–Broadcast), and meteorological data. The ISO 20906 standard [17] defines a threshold-based method for separating aircraft noise events, but its accuracy is limited by background noise (such as traffic and industrial activities). Meteorological data (such as wind speed, wind direction, and temperature) can influence the characteristics of noise propagation. For instance, wind speed and direction can affect the distance and direction of sound propagation. ADS–B data can provide information on the aircraft’s position, speed, and flight path. By aligning the timestamps of ADS–B data with acoustic data, the flight trajectories of aircraft can be correlated with the time and location of noise events, thereby aiding in the localization of noise sources. The low cost of obtaining ADS–B data, which can cover almost all aircraft trajectories, departure times, and arrival times, offers a new method for efficiently and cost-effectively constructing airport noise maps [18].

Nonetheless, the effective integration of multi-source data, the establishment of models for the spatiotemporal propagation patterns of noise, and the quantification of environmental impacts remain the primary challenges in the current research domain. By integrating various data sources, including noise data, meteorological data, and flight information, researchers can more comprehensively analyze the spatiotemporal distribution characteristics of airport noise. Spatial distribution modeling techniques provide crucial support for the refined management and decision-making processes of airport noise, for instance, generating noise distribution maps using spatial interpolation techniques based on limited noise sampling points [19]. Similarly, the computation and spatial visualization of road traffic noise can also be achieved through spatial interpolation methods [20], which offers valuable references for the refined management of airport noise. However, several limitations still exist in the field of airport noise monitoring technology, including the irrational layout of monitoring sites, the insufficient performance of monitoring equipment, and the limitations in data processing and analysis capabilities. Moreover, the issue of spatial interpolation accuracy for sparse monitoring sites has not been fully resolved, and the applicability of methods such as Inverse Distance Weighting (IDW) and Kriging in complex airport environments still requires further validation [21,22]. Specifically, the IDW method simplifies the calculation process through distance-weighted averaging, while the Kriging method captures spatial autocorrelation through the semivariogram [23,24,25].

Despite the extensive application of Inverse Distance Weighting (IDW) and Kriging interpolation methods in fields such as geology, their utilization in airport noise monitoring remains relatively restricted. This investigation explores the application of multi-source data fusion technology, integrating noise data, meteorological data, and ADS–B flight data, to achieve not only precise noise-event attribution and refined management but also the construction of a comprehensive framework for airport noise monitoring. Through the fusion of multi-source data, correlation analyses between noise data and meteorological and flight data are conducted, leading to the establishment of a multi-source data fusion model for the spatial distribution of airport noise. Subsequently, a spatial distribution model for airport noise is established, enabling refined monitoring, precise tracing, and management of noise, and enhancing the utilization efficiency of noise monitoring data.

## 2. Materials and Methods

### 2.1. Sensor-Related Technology

Environmental noise monitoring sensors serve as instruments for the real-time measurement and recording of environmental noise levels [26]. The acoustic sensor constitutes the core component of these devices, typically detecting fluctuations in sound pressure via the piezoelectric effect or alterations in capacitance. Piezoelectric sensors transduce sound pressure into electrical signals by leveraging piezoelectric materials such as quartz crystals or ceramics, whereas capacitive sensors discern sound pressure through the measurement of capacitance variations induced by sound waves. The operational mechanism primarily entails the sensor’s acoustic front end capturing ambient sound waves, which in turn instigate changes in the piezoelectric material or capacitance within the sensor, thereby engendering electrical signals. Subsequently, these electrical signals undergo amplification and filtering to excise extraneous noise and interference. The processed signals are then digitized and either stored within the sensor’s storage unit or conveyed to a data processing center via a wireless communication module.

Meteorological monitoring sensors, as a vital component of the airport noise monitoring system, are capable of real-time collection of multiple meteorological parameters, including wind speed, wind direction, air temperature, atmospheric pressure, relative humidity, and precipitation [27]. These parameters are essential for analyzing the environmental impact on noise propagation, as meteorological conditions can significantly influence the path and intensity of noise. Specifically, wind speed and wind direction sensors, which typically employ cup-type or propeller-type anemometers and wind vanes, provide real-time data on wind speed and direction by measuring the horizontal and vertical components of the wind. Air temperature and atmospheric pressure sensors, based on the principles of thermistors or pressure-sensitive resistors, can accurately measure ambient temperature and atmospheric pressure. Relative humidity sensors, which measure the water vapor content in the air, provide data on relative humidity. Humidity can affect the absorption and scattering of sound waves, thereby influencing the propagation distance and intensity of noise [28]. Precipitation sensors, usually using tipping-bucket or optical rain gauges, can monitor precipitation in real time.

Automatic Dependent Surveillance–Broadcast (ADS–B) is an aviation surveillance technology that enables aircraft to broadcast their positions and other flight information automatically. ADS–B sensors are capable of real-time reception and decoding of ADS–B signals transmitted by aircraft [29,30]. These signals encompass critical information such as the aircraft’s real-time location, altitude, speed, and heading, characterized by a high data update frequency and reliable accuracy. ADS–B sensors operate at the 1090 MHz frequency, facilitating efficient and cost-effective surveillance of flights without reliance on ground-based radar. Owing to their high precision and real-time capabilities, ADS–B sensors are extensively utilized in air traffic management, flight tracking, and airport operations optimization. They provide essential dynamic flight information support for multi-source data fusion.

The Internet of Things (IoT) represents a technological paradigm that facilitates the real-time accumulation of data for objects or processes that require monitoring, connection, and interaction. This is achieved through the deployment of information sensors, Radio Frequency Identification (RFID) technology, Global Positioning System (GPS), and a variety of other devices and technologies [31]. The collection of critical information such as sound, light, heat, electricity, and location, when connected through diverse networks, realizes ubiquitous connections between objects and between objects and people. This, in turn, establishes intelligent perception, identification, and management of goods and processes. The IoT, often referred to as the “Internet of Everything”, enables seamless interconnectivity and communication between people, machines, and objects, anytime and anywhere. With the ongoing advancement of IoT and sensor technologies, an increasing number of IoT sensor platforms are being widely applied across various domains.

### 2.2. Multi-Source Data Fusion

In the aviation industry, the accuracy of noise monitoring hinges not only on the precision of individual data sources but crucially on the holistic integration and analysis of data from multiple sources. This research, by integrating noise data, meteorological data, and ADS–B flight data, has meticulously examined the spatiotemporal distribution patterns of airport noise, thereby markedly improving the accuracy and credibility of the monitoring process. The temporal correlation between noise data and meteorological data serves as the cornerstone of integrated data fusion techniques. Meteorological conditions such as wind speed, wind direction, temperature, and humidity exert a substantial influence on the propagation and attenuation of noise. Employing signal processing and analysis methods facilitates the preprocessing of meteorological data to extract time-series information. The temporal correlation between noise data and ADS–B flight data, particularly when an aviation noise event transpires, enables the correlation of noise data with ADS–B flight data through temporal association. ADS–B data divulges critical information regarding the real-time location, speed, and altitude of aircraft, which is indispensable for tracking and analyzing noise events. Through timestamp matching, a connection between specific noise events and specific flights can be established, thereby enabling precise tracking of noise events.

Specifically, this study meticulously ensured the high quality and usability of the data through a series of data preprocessing steps, including data cleaning, noise reduction, and feature extraction. Utilizing timestamps as the linking key, noise data are correlated with meteorological and ADS–B data through the application of data synchronization and time calibration techniques. For each noise event, ADS–B data with matching timestamps are identified to ascertain the aircraft’s position and flight status. Noise Event Tracing, in conjunction with meteorological and ADS–B data, analyzes the propagation path and impact range of noise events. Through integrated data fusion, precise tracking of noise events is achieved, thereby providing scientific support for noise management.

Moreover, multi-source data fusion technology can also support the quantitative assessment of noise impacts. By integrating noise prediction results with noise impact evaluation criteria, the degree of noise impact on surrounding residents and the environment can be quantified. The application of the multi-source data fusion technology introduced in this study to airport noise monitoring not only furnishes airport managers with scientific evidence to devise effective noise mitigation strategies, thereby alleviating the adverse impact of noise on surrounding communities, but also markedly boosts the sustainability of airport operations. The novelty and practicality of this technological approach bear substantial theoretical and practical significance for the field of airport noise management.

### 2.3. Spatial Distribution Modeling

Spatial distribution modeling of airport noise, as a pivotal component of monitoring technology, can effectively elucidate the distribution characteristics of noise in the areas surrounding airports. Geostatistical models possess the capability to assess noise levels in specific regions and delineate the spatiotemporal dynamics of noise pollution [32], thereby furnishing a scientific basis for noise control and decision-making. In the realm of spatial distribution modeling, prevalent techniques encompass Kriging interpolation, Inverse Distance Weighting (IDW), and multiple regression models. The resultant spatial distributions derived from these methods can be effectively visualized on Geographic Information System (GIS) platforms, thereby augmenting their interpretability [33]. Despite the extensive utilization of IDW and Kriging interpolation in domains such as geology, their application within the context of airport noise monitoring has been somewhat restricted. This study introduces a novel hybrid approach that integrates IDW and Kriging interpolation, specifically tailored for airport noise monitoring, with the aim of enhancing the precision and computational efficiency of the interpolation process. The implementation of this hybrid method represents a significant innovation in airport noise monitoring, particularly when addressing sparse monitoring data, as it enables a more accurate depiction of noise spatial distribution patterns. This study will concentrate on Kriging interpolation and Inverse Distance Weighting (IDW).

#### 2.3.1. Inverse Distance Weighting (IDW)

Inverse Distance Weighting (IDW) is a classic and widely used spatial interpolation method, grounded in Tobler’s first law of geography, which posits that “everything is related to everything else, but near things are more related than distant things”. This method assigns weights to known data points based on their spatial distance from the interpolation point, with closer points receiving higher weights. It then performs a weighted average interpolation. The core concept of the IDW method is that known points closer to the prediction point exert a greater influence on the predicted value, while those farther away have a relatively smaller influence.

Specifically, the Inverse Distance Weighting (IDW) method assigns weights to each known data point based on the inverse of the distance from the interpolation point. The weights are inversely proportional to the distance, meaning that points closer to the interpolation point are given higher weights, while those farther away are given lower weights. This weighting scheme ensures that the influence of known points diminishes rapidly with increasing distance. IDW is particularly well-suited for terrain modeling and geographic data interpolation because it can generate smooth surfaces and is computationally efficient. However, IDW is sensitive to outliers and may not capture complex spatial patterns effectively. In practice, IDW is widely used in Geographic Information Systems (GIS) to generate continuous surfaces for geospatial data.

To conduct IDW interpolation, the initial step involves identifying the coordinates and extent of the interpolation points, as well as the coordinates and associated values of the known sampling points. Following this, the interpolation is executed by computing the weights and subsequently applying a weighted average [34]. For each interpolation point, the distances to all known sampling points are determined and then converted into corresponding weight values. The magnitude of these weights signifies the contribution of each known sampling point to the predicted value, with the weights being inversely proportional to the distance. Typically, the calculation of weights is based on Equation (1).

(1) $$w_{i}=\frac{1}{d_{i}^{p}}$$

where $w_{i}$ is the weight of the i-th sample point, $d_{i}$ is the distance between the sample point and the interpolation location, and p is an adjustable parameter, typically set to two (Euclidean distance) or three (Manhattan distance), to control the rate of weight decay with distance. Additionally, the optimal value of p can also be determined through methods such as cross-validation.

After calculating the weights for each known sample point, the function values are combined through a weighted average to obtain the function value at the interpolation point. The specific calculation process can be referred to in Equation (2).

(2) $$z(x,y)=\frac{\sum_{i=1}^{N}w_{i}z_{i}}{\sum_{i=1}^{N}w_{i}}$$

where N is the number of known sample points, and $z_{i}$ is the function value of the i-th sample point.

Subsequently, a maximum search radius is defined, and only the known points within this radius are utilized for interpolation calculations. This approach reduces the computational load and enhances local accuracy. By setting the search neighborhood, the computation time is shortened, and data points that have a negligible impact on the prediction results are excluded. The strategy of limiting the number of measurements, that is, by specifying the search neighborhood, is a widely adopted approach. The geometric shape and parameters of the search neighborhood can be appropriately adjusted according to the characteristics of the data distribution and the influence of anisotropy.

#### 2.3.2. Kriging Interpolation

Kriging interpolation, as a geostatistical spatial interpolation technique, is designed to fully account for the spatial autocorrelation between data points, thereby generating a prediction surface that is both scientifically rigorous and realistically reflective of spatial variations. This method quantifies spatial autocorrelation through the variogram, a fundamental tool in geostatistics that describes how the spatial continuity of the data varies with distance. By leveraging the variogram, Kriging employs a linearly weighted average of known point values to predict unknown point values, thus, constructing an interpolation model that minimizes estimation variance. This approach not only provides an optimal prediction but also offers an associated measure of uncertainty, making Kriging a powerful tool for spatial analysis [35].

In the realm of airport noise monitoring, Kriging interpolation can effectively estimate noise distribution in areas surrounding airports using limited noise monitoring site data. Through the variogram modeling process, Kriging interpolation provides an assessment of the uncertainty associated with noise predictions, specifically the Kriging variance. This feature endows Kriging interpolation with a significant advantage when dealing with data that exhibit spatial autocorrelation.

The fundamental assumption of Kriging interpolation is that the spatial attribute z is stationary, meaning that for any point in space (x,y), there is the same expectation c and variance $\sigma^{2}$. Thus, Kriging interpolation is based on a set of assumptions, including that the statistical properties of the data (such as the mean and variance) remain constant across space. Moreover, spatial autocorrelation is also assumed, that is, the values of adjacent points are more similar to each other than those of distant points, thereby reflecting the continuity characteristic of spatial data. This type of autocorrelation can be quantified through the variogram.

The variogram is a fundamental tool in Kriging interpolation, primarily used to quantify the spatial autocorrelation of data. It measures how the variance of the differences between data points changes with the distance separating them. The variogram is mathematically expressed in Equation (3).

(3) $$\gamma (h)=\frac{1}{2N(h)}\sum_{i=1}^{N(h)}[z(x_{i})-z(x_{i}+h){]}^{2}$$

where γ(h) is the semivariance, indicating the degree of variation between point pairs separated by a distance h; h represents the distance (lag distance) between point pairs; $z(x_{i})$ and $z(x_{i}+h)$ are the observed values at locations $x_{i}$ and $x_{i}+h$, respectively. N(h) is the number of point pairs separated by a distance h.

Several key aspects of the variogram include the nugget effect, which is the nonzero semivariance when h approaches zero, indicating measurement error or microscale variation; the sill, which is the value at which the semivariance stabilizes as h increases, representing the overall variability of the data; and the range, which is the distance at which the semivariance reaches the sill, indicating the maximum extent of spatial autocorrelation.

Ultimately, the mathematical model for Kriging interpolation is constructed, which processes the values of known points through a linearly weighted average to predict the values at unknown points. The specific mathematical expression of the model is presented in Equation (4).

(4) $$\hat{z}(x_{0})=\sum_{i=1}^{n}\lambda_{i}z(x_{i})$$

where $\hat{z}(x_{0})$ is the predicted value at the unknown location $x_{0}$,$z(x_{i})$ is the observed value at the known location $x_{i}$, and $\lambda_{i}$ is the weight coefficient, indicating the contribution of the known point $x_{i}$ to the predicted value $\hat{z}(x_{0})$.

To ensure that the estimate $\hat{z}(x_{0})$ is unbiased, the constraints satisfied by the weighting coefficient $\lambda_{i}$ is presented in Equation (5).

(5) $$\sum_{i=1}^{n}\lambda_{i}=1$$

The objective of kriging interpolation is to minimize the variance of the estimation error, that is, to minimize $\sigma^{2}$. The calculation of $\sigma^{2}$ is presented in Equation (6).

(6) $$\sigma^{2}=Var[\hat{z}(x_{0})-z(x_{0})]$$

By solving the optimization problem presented above, the optimal weight coefficient $\lambda_{i}$ can be derived, which minimizes the variance of the estimation error, thus, completing the interpolation calculation.

Therefore, the Kriging interpolation method involves multiple steps, including data preparation, variogram modeling, weight calculation, and interpolation. Specifically, the spatial locations and observed values of known sampling points must first be collected. Subsequently, the experimental variogram is calculated and fitted to an appropriate theoretical variogram model (such as the spherical model, exponential model, or Gaussian model). Based on the established variogram model, the weight coefficients $\lambda_{i}$ are further solved. Using these weight coefficients and the observed values of the known points, the predicted values of the unknown points can be calculated, and the variance of the prediction error can be estimated to assess the reliability of the interpolation results.

### 2.4. Noise Theoretical Methods

To select appropriate noise monitoring indicators for this study, relevant standards [17] were consulted to ensure that the chosen metrics would provide a comprehensive reflection of the characteristics and impacts of airport noise. During the noise calculation and evaluation process, several key metrics were deemed particularly important [36,37,38]. These included the equivalent continuous sound level ($L_{eq}$), maximum sound level ($L_{\max}$), and minimum sound level ($L_{\min}$), as well as the exposure levels associated with aviation noise events and calculations for individual noise events. Specifically, $L_{eq}$ serves as a measure of the average noise level over a specified period and provides an indication of the overall energy of the noise. $L_{\max}$ and $L_{\min}$ are utilized to assess the peak and background levels of noise, respectively, which facilitates the identification of impulsive noise events and continuous noise. Additionally, cumulative percentile sound levels, such as $L_{10}$, $L_{50}$, and $L_{90}$, offer insights into the distribution of noise across different time percentiles, thereby assisting in the evaluation of noise persistence and variability.

$L_{AE}$ (Exposure Sound Level): Exposure sound level refers to the A-weighted sound pressure level equivalent to a duration of 1 s for a specified measurement period or a single independent noise event. It is used to assess the total energy of a noise event and is an important indicator for measuring the degree of noise exposure.

$L_{eq}$ (Equivalent Continuous Sound Level): Equivalent continuous sound level refers to the time average of the sound pressure level over a specified time interval. It converts the sound pressure levels at different moments into an equivalent continuous sound pressure level through energy averaging, which is used to represent the average noise level over a period of time.

$L_{\max}$ (Maximum Sound Level): Maximum sound level refers to the maximum A-weighted sound level measured during a specified measurement period or for a single independent noise event. It reflects the highest noise level in a noise event and is very important for assessing the peak impact of noise.

$L_{\min}$ (Minimum Sound Level): Minimum sound level refers to the minimum A-weighted sound level measured during a specified measurement period or for a single independent noise event. It reflects the lowest noise level in a noise event and is of great significance for assessing the lower-limit impact of noise.

$L_{10}$ (10% Exceeded Sound Level): The 10% exceeded sound level is the A-weighted sound pressure level exceeded for 10% of the time during the considered period T. It indicates that during the measurement period, the noise level is higher than this value for 10% of the time and is used to assess the distribution of higher noise levels.

$L_{50}$ (Median Sound Level): The median sound level is the A-weighted sound pressure level exceeded for 50% of the time during the considered period T. It indicates that during the measurement period, the noise level is higher than this value for 50% of the time and represents the median of the noise level.

$L_{90}$ (90% Exceeded Sound Level): The 90% exceeded sound level is the A-weighted sound pressure level exceeded for 90% of the time during the considered period T. It indicates that during the measurement period, the noise level is higher than this value for 90% of the time and is used to assess the distribution of lower noise levels.

SEL (Sound Exposure Level): Sound exposure level refers to the integral of the squared sound pressure over a specified time period or an event of duration T, calculated according to a formula. It is used to assess the total energy of a noise event and is similar to exposure sound level but with a different calculation method.

**Figure 1:**
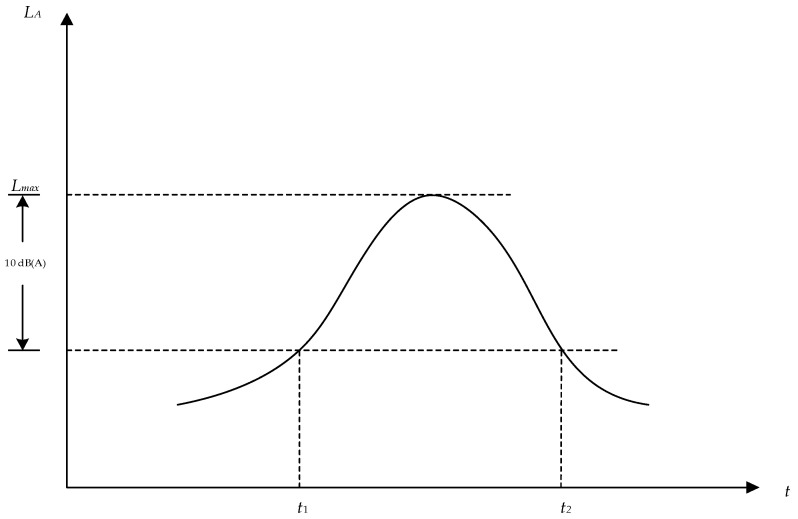
Schematic diagram of a single noise event.

Single Aviation Noise Event: For a single noise event, the exposure sound level, maximum sound level, start time of the noise event $t_{1}$, end time $t_{2}$, and duration $T_{c}(s)$ should be measured. The integration time for the exposure sound level is the duration $T_{c}(s)$ from the start to the end below 10 dB(A) of the maximum sound level of the single noise event, i.e., $T_{c}=t_{2}-t_{1}$, as shown in Figure 1.

The formula for calculating the exposure sound level is presented in Equation (7).

(7) $$L_{AE}=10\times \mathrm{lg}\left(\frac{1}{T_{0}}\int_{t_{1}}^{t_{2}}10^{0.1\times L_{A}}dt\right)$$

where $L_{AE}$ is the exposure sound level for a single noise event and $L_{A}$ is the instantaneous A-weighted sound level at time t. $t_{1}$ is the start time of the single noise event and $t_{2}$ is the end time of the single noise event. $T_{0}$ represents 1 s.

## 3. System Architecture

### 3.1. Noise Monitoring Framework

**Figure 2:**
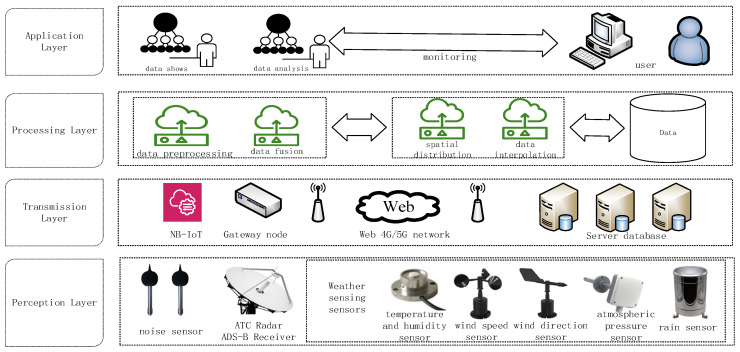
Schematic diagram of the airport noise monitoring architecture.

To achieve the objective of noise monitoring around airports, it is necessary to establish several noise monitoring stations at strategically selected locations around the airport and deploy noise sensors. Additionally, meteorological data, including temperature and humidity, as well as flight data, should be collected. By integrating multi-source data fusion, spatial distribution modeling, and intelligent analysis techniques, efficient perception and monitoring of airport noise can be realized. The overall architecture of airport noise monitoring is depicted in Figure 2 and can be divided into four layers: the perception layer, the transmission layer, the processing layer, and the application layer.

#### 3.1.1. Perception Layer

The setup encompasses noise sensors, weather sensors, and ADS–B receivers strategically placed around the airport. Noise sensors are tasked with capturing sound pressure level data, weather sensors with recording temperature, humidity, wind speed, and wind direction data, and ADS–B receivers with obtaining flight position, altitude, and speed information. All sensors transmit data to a central gateway via low-power wireless communication modules (e.g., LoRa or NB-IoT). This configuration forms the foundation for real-time collection of noise, weather, and ADS–B flight data at the airport. The specific equipment configuration is detailed as follows:

Noise Sensors: The sound pressure level detection sensors employed in this study are characterized by high precision, featuring a measurement range spanning from 30 to 130 dB and a frequency response range of 20 Hz to 20 kHz. To ensure accurate capture of instantaneous noise fluctuations, the data acquisition frequency is set at 1 Hz (i.e., one measurement per second), with a measurement error maintained within ±3%. For optimal performance, the installation height of the detection equipment should exceed 1.2 m and be positioned at least 3.5 m away from all reflective surfaces (excluding the ground) to minimize interference from reflected noise on the measurement results. The detection equipment is interfaced with the data acquisition unit via an RS485 connection to facilitate real-time data transmission.

Weather Sensors: This category encompasses a variety of sensor types, including temperature sensors, humidity sensors, anemometers, and wind vanes, which are capable of real-time monitoring of key meteorological parameters such as wind speed, wind direction, temperature, humidity, air pressure, and precipitation. These parameters are crucial for analyzing noise propagation and its attenuation mechanisms. The weather sensors utilize ultrasonic probes for measuring wind speed and direction, and their design, which lacks moving parts, ensures the high reliability of the data. The temperature sensor has a measurement range spanning from −40 °C to 85 °C, the humidity sensor covers a range of 0% to 100% relative humidity, and the wind speed sensor is capable of measuring wind speeds from calm conditions up to 60 m/s.

ADS–B Flight Data Acquisition System: Operating at the 1090 MHz frequency band, this system is capable of real-time acquisition of key flight information such as position, velocity, and altitude, with a data update rate of 1 Hz, thereby ensuring the immediacy of flight dynamic information. The collected data is transmitted to the data acquisition unit via a wireless communication module, providing robust data support for the tracking and analysis of noise events.

#### 3.1.2. Transmission Layer

This layer is composed of multiple gateway nodes, which are responsible for receiving data from the perception layer and transmitting it to the remote control center. The gateway nodes establish connections with the remote control center via 4G/5G networks or fiber optics to ensure the real-time and reliable transmission of data. The network architecture for airport noise monitoring adopts a distributed design to ensure real-time data transmission and processing. The specific network architecture is as follows:

Sensor Network: Noise sensors, weather sensors, and the ADS–B data acquisition system are connected to the data acquisition unit via wireless communication modules. The sensor network adopts a star topology to ensure the stability and reliability of data transmission.

Data Transmission Network: The data acquisition unit transmits data to the central data processing center via Ethernet or 4G/5G networks. The data transmission network is designed with redundancy to ensure high availability of data transmission.

Data Processing Center: The central data processing center is responsible for the fusion, analysis, and storage of the collected data, ensuring the efficiency of data processing and the security of data storage.

#### 3.1.3. Processing Layer

Data preprocessing and multi-source data fusion are implemented for the collected data. Based on the fused data, event correlation is performed in accordance with the principle of timestamp alignment. Spatial distribution modeling techniques are employed to conduct interpolation of noise monitoring sites, thereby establishing a noise distribution model to aid in environmental impact assessment. The processed noise data, meteorological data, and ADS–B data provide a solid foundation for subsequent data analysis.

#### 3.1.4. Application Layer

Primarily designed to present a graphical interface of noise monitoring data, it visually displays noise distribution and offers functions for data retrieval and analysis. This layer provides auxiliary information for the decision-making process in noise management, enabling stakeholders to make informed decisions based on the presented data.

### 3.2. Data Acquisition and Network Transmission

**Figure 3:**
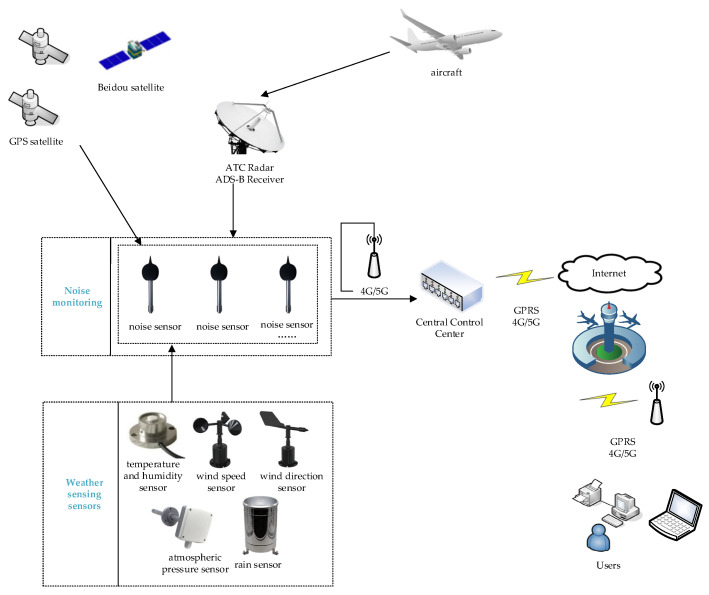
Schematic diagram of the network transmission architecture.

Following the acquisition of data by noise and other sensors, the information is conveyed to the control center through the Internet of Things and 4G/5G communication technology networks for subsequent data processing. The specific network architecture is depicted in Figure 3.

Specifically, noise sensors, weather sensors, and ADS–B receivers are tasked with real-time data collection and subsequently transmit the data to the gateway nodes via Internet of Things (IoT) modules. The gateway nodes package the received data and relay it to the control center via 4G/5G networks. At the control center, raw data undergo noise reduction, missing value imputation, and format normalization to ensure high-quality data for subsequent analysis. In the subsequent analysis, noise data, weather data, and flight trajectory data are synchronized in time and aligned in space to generate a multi-source integrated dataset, thereby facilitating the correlation of relevant events.

## 4. Results

### 4.1. Site Layout

The impact range of aviation noise is influenced by a variety of factors, including the take-off and landing status of aircraft and changes in flight altitude. Noise-sensitive buildings that are close to the airport and near the projection lines of flight routes are more significantly affected by aviation noise. By measuring the distance between the measurement points and the projection lines of the flight routes or the airport, it can be determined which locations are more affected by aviation noise. Therefore, monitoring sites are strategically set up near these noise-sensitive buildings.

When selecting monitoring sites, several principles should be adhered to in order to ensure the accuracy and reliability of noise measurements. Firstly, sites should be chosen to avoid proximity to roads and fixed equipment that may generate extraneous noise. Additionally, they should be situated away from areas with frequent human activity to minimize background noise interference. Ideally, locations with lower background noise levels should be selected to enhance the clarity of aviation noise data. The layout of monitoring sites must also consider the safety of instruments and equipment, as well as the convenience for personnel maintenance. This includes ensuring the safety of both instruments and personnel, and the accessibility of maintenance work. Furthermore, the surrounding environment of the monitoring sites should meet the requirement of being “open and flat” to ensure that measurement personnel can clearly observe the main parts of the flight routes.

Given that noise intensity is inversely proportional to the distance from the source, the distance between noise-sensitive buildings and flight routes can serve as a basis for assessing the degree of noise impact. The attenuation of sound level from a point source with distance is shown in Equation (8).

(8) $$L(r)=L(r_{0})-20\mathrm{lg}(r/r_{0})$$

where L(r) represents the sound pressure level at the measurement point and $L(r_{0})$ is the sound pressure level at the reference position; r is the distance from the measurement point to the sound source and $r_{0}$ is the distance from the reference position to the sound source.

**Figure 4:**
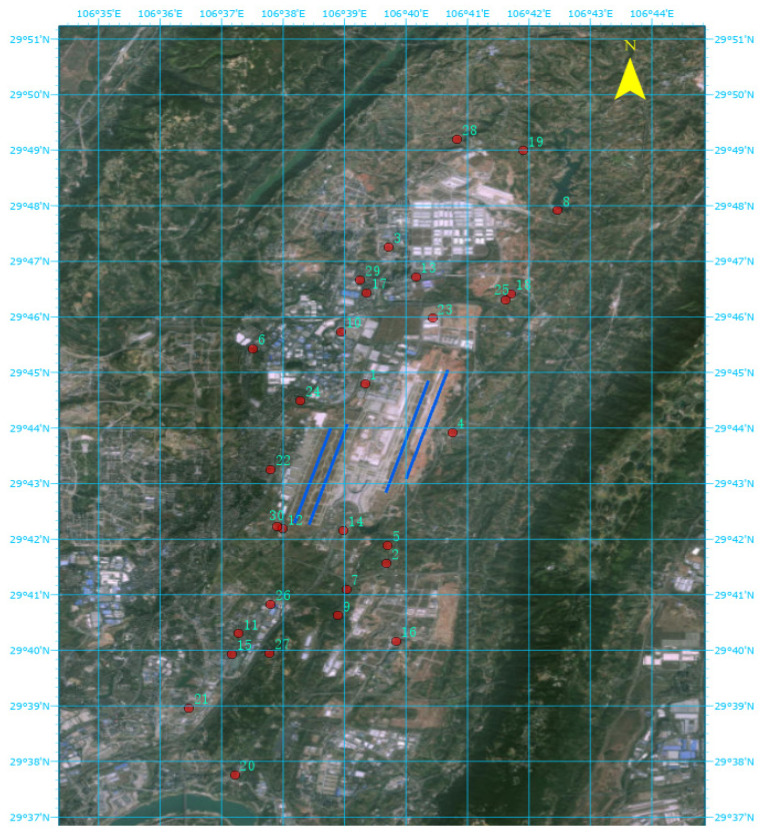
Distribution Map of Noise Monitoring Stations.

This research project was implemented in the vicinity of Chongqing Jiangbei International Airport in China. In accordance with the site layout standards, thirty noise monitoring stations were established in key areas along the airport’s flight routes, including industrial parks, residential communities, schools, and parks. The specific locations of the monitoring sites are shown in Figure 4, where the red dots represent the monitoring stations, and the station numbers are labeled beside them. The installation height of the noise sensors was determined based on the actual conditions of each monitoring sitet. Eight monitoring sites were installed on 4.5-m-high poles, while the remaining 22 monitoring sites were installed on building rooftops at a height of 1.5 m.

### 4.2. Data Acquisition and Analysis

As detailed in Section 2.4, parameters such as $L_{eq}$, $L_{\max}$, and $L_{\min}$ are commonly used to describe noise conditions in the field of noise monitoring. Noise sensors continuously monitor noise levels at each monitoring station and store the corresponding datasets. For this study, specific dates’ monitoring data were selected for analysis. In accordance with the requirements of the Civil Aviation Industry Standard of the People’s Republic of China MH/T5109-2013, “Technical Specification for Airport Aircraft Operation and Noise Monitoring System”, hourly reports of aviation noise are generated. Specifically, this study extracted monitoring data from 00:00:00 to 23:59:59 on 9 January 2025, covering 24 h of noise and meteorological data for that day. The hourly reports include hourly airport noise and meteorological data from each monitoring station for the day. Additionally, the study integrates noise event data and ADS–B flight data to conduct a comprehensive analysis of airport noise and aviation events.

Given the large volume of data from each monitoring station, this study takes Noise Monitoring Station 1 as an example to present the hourly monitoring data for 9 January 2025. See Table 1 for details.

**Figure 5:**
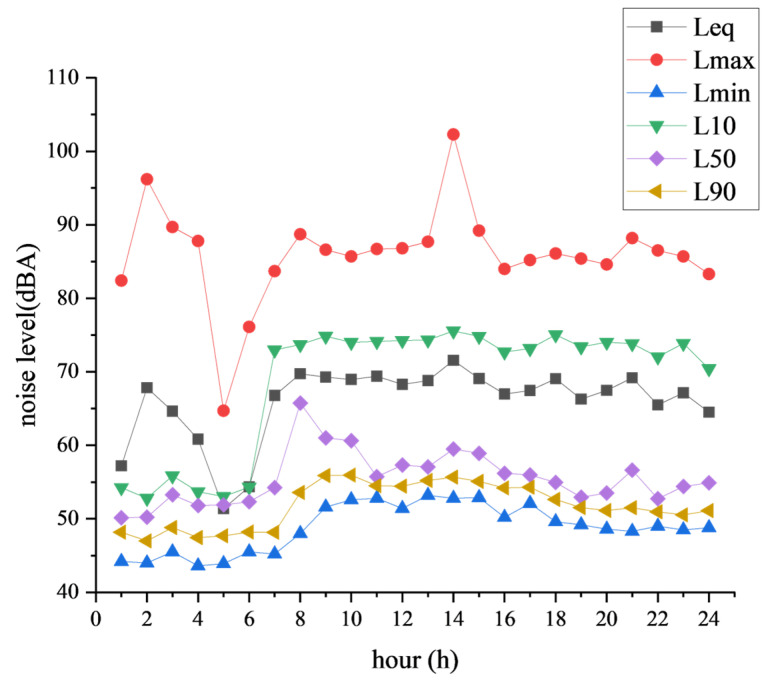
Hourly noise monitoring chart for Noise Monitoring Site 1 on 9 January 2025.

In accordance with the requirements, detailed records were maintained for the hourly $L_{eq}$ (equivalent continuous sound level), $L_{\max}$ (maximum sound level), $L_{\min}$ (minimum sound level), $L_{10}$ (sound level exceeded 10% of the time), $L_{50}$ (median sound level), and $L_{90}$ (sound level exceeded 90% of the time) on the day of the study. The specific data are shown in Figure 5.

**Figure 6:**
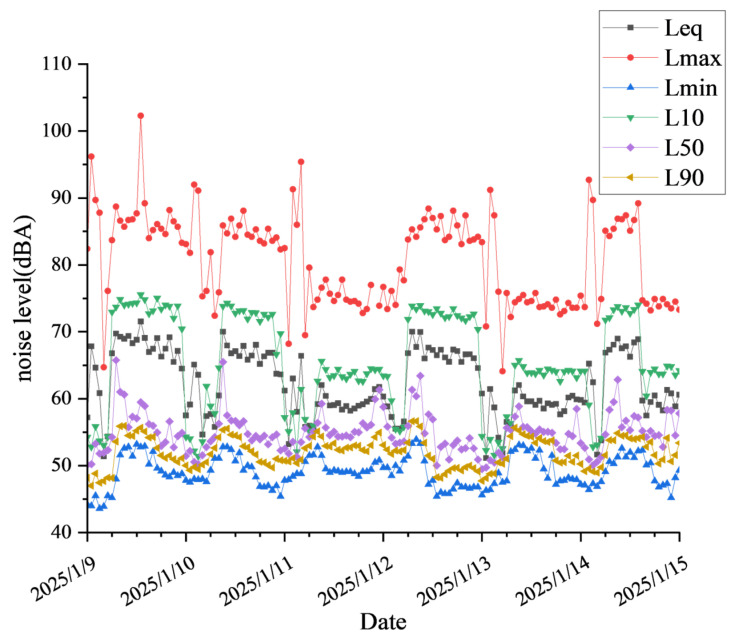
Noise monitoring chart for Noise Monitoring Site 1 from 9–15 January 2025.

To elucidate the long-term trends in noise monitoring, we have incorporated a noise monitoring data chart for Noise Monitoring Site 1, covering the period from 9–15 January 2025, as depicted in Figure 6.

To ensure the accuracy of the data, noise measurements should be conducted under stable meteorological conditions, specifically in the absence of precipitation or thunderstorms. The measurements should be carried out in environments where the wind speed does not exceed 10 m per second. The wind speed at the monitoring sites must be measured on-site. Interference from the sounds of rain and thunder can increase the background noise level, thereby affecting the accuracy of the monitoring data. In addition, meteorological phenomena such as rain, snow, and thunderstorms may cause flight delays or cancellations, which in turn can lead to fluctuations in the average number of take-offs and landings per day at the airport, affecting the results of aviation noise monitoring. Therefore, it is necessary to collect meteorological monitoring data, which includes wind speed, wind direction, temperature, air pressure, relative humidity, and precipitation, to facilitate subsequent data fusion and analysis. The specific meteorological data are presented in Table 2.

To investigate the noise levels at different sites within the same time interval, this study collected equivalent noise data from all sites at specific hours. Specifically, the equivalent noise values from the thirty sites at 12:00 on 9 January 2025, were selected for analysis. The detailed data are presented in Table 3.

**Figure 7:**
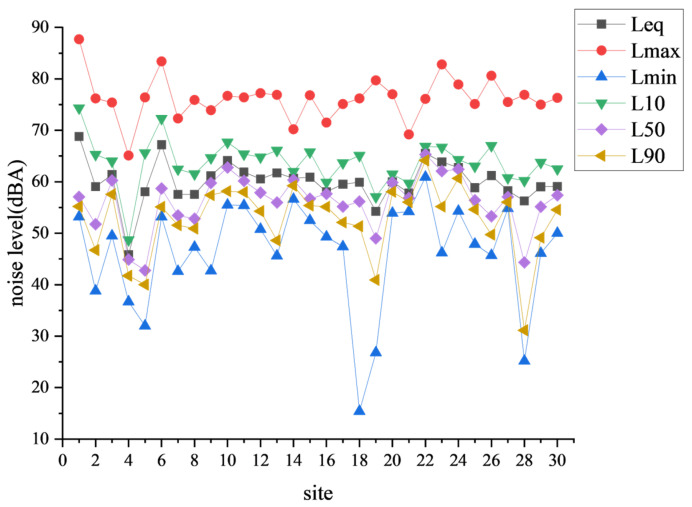
Chart of hourly equivalent values at 12:00 for thirty monitoring sites.

A comprehensive comparative analysis was conducted on the various monitoring indicators of the thirty noise monitoring stations during this time period. The results are depicted in Figure 7.

### 4.3. Noise Event Correlation

**Figure 8:**
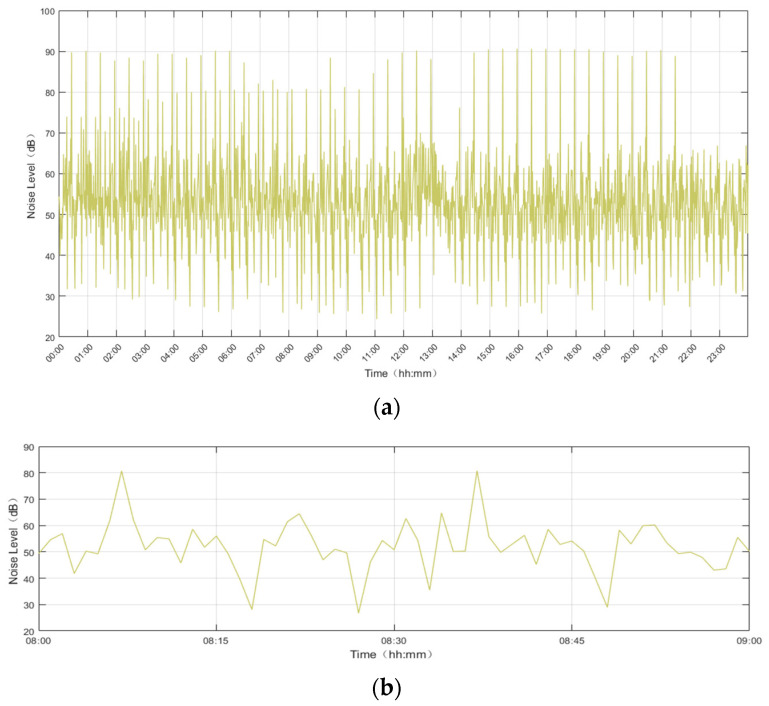
Detailed 24-h noise monitoring data chart for Noise Monitoring Site 1 on 9 January 2025: (**a**) 24-h monitoring curve chart for Monitoring Site 1; (**b**) Enlarged view of the monitoring curve.

Correspondingly, data from all monitoring stations at various time intervals were recorded and subjected to comprehensive analysis. Through this approach, noise events during each time period could be analyzed in detail, and daily noise events could be summarized. When noise events occurred, they could be correlated with flight information. Taking Monitoring Station 1 as an example, this study presents detailed monitoring data for the entire 24 h of 9 January 2025, with some data being magnified for better illustration, as shown in Figure 8.

To further enhance the understanding of noise events, this study conducted an in-depth analysis of noise events within specific time periods, in conjunction with Automatic Dependent Surveillance–Broadcast (ADS–B) flight data. This integration allowed for the differentiation between aviation and non-aviation noise. For aviation noise, correlation analysis with flight data enabled the identification of specific flights causing noise events and the tallying of aircraft types for each flight on that day. This approach facilitated a quantitative analysis of the frequency of noise events associated with different aircraft types.

Taking the data from Noise Monitoring Station 1 on January 9 as an example, given the large number of noise events on that day, this study selected 10 consecutive events for detailed analysis. The specific data are shown in Table 4.

For noise events, through the analysis of timestamps in conjunction with Automatic Dependent Surveillance–Broadcast (ADS–B) data, it can be further determined whether they fall within the category of aviation noise. If confirmed as an aviation noise event, additional information such as flight number, flight trajectory, and aircraft type can be correlated. Taking the data in row 5 of Table 4 as an example (other noise events can also be processed by this method), the start time of this event is 12:15:14, the end time is 12:15:36 and the duration is 49 s. The $L_{eq}$ is 74.37 dBA, the SEL is 86.41 dBA, and the $L_{\max}$ is 82.00 dBA. According to ADS–B data, this event is associated with flight number 3U3209, which is an Airbus A350-941.

**Figure 9:**
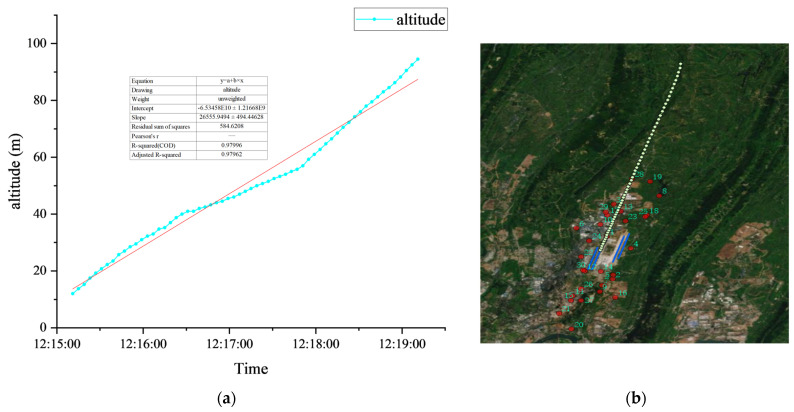
Flight path chart for Flight 3U3209 during takeoff: (**a**) Take-off altitude of the track varying with time; (**b**) The distribution of the track in the horizontal plane.

For the specific flight event, namely flight 3U3209, its flight trajectory is a take-off track. Further analysis of this take-off track data is shown in Figure 9, where the plane (a) illustrates the take-off altitude of the track varying with time, and the plane (b) presents the distribution of the track in the horizontal plane. The red dots denote the locations of the noise monitoring sites, with the corresponding numbers indicating the site identifiers.

### 4.4. Spatial Distribution Model

#### 4.4.1. Pre-Monitoring Data

Accurate identification and matching of noise events with aircraft operation-related data are of great significance for the traceability of aviation noise data and the conduct of monitoring work. By arguing the representativeness of the data and the credibility of the calculation model, noise levels over a longer time span or in other unmonitored sensitive areas can be analyzed and calculated. Although thirty noise monitoring stations have been established, given that they cannot fully cover the entire area, interpolation methods can be used to construct spatial distribution models. For specific flight events, taking flight 3U3209 as an example, the $L_{eq}$ equivalent values of each monitoring station at 12:15 on 9 January 2025, were selected for further analysis (the analysis method for data at other time points is the same). The specific data are shown in Table 5.

**Figure 10:**
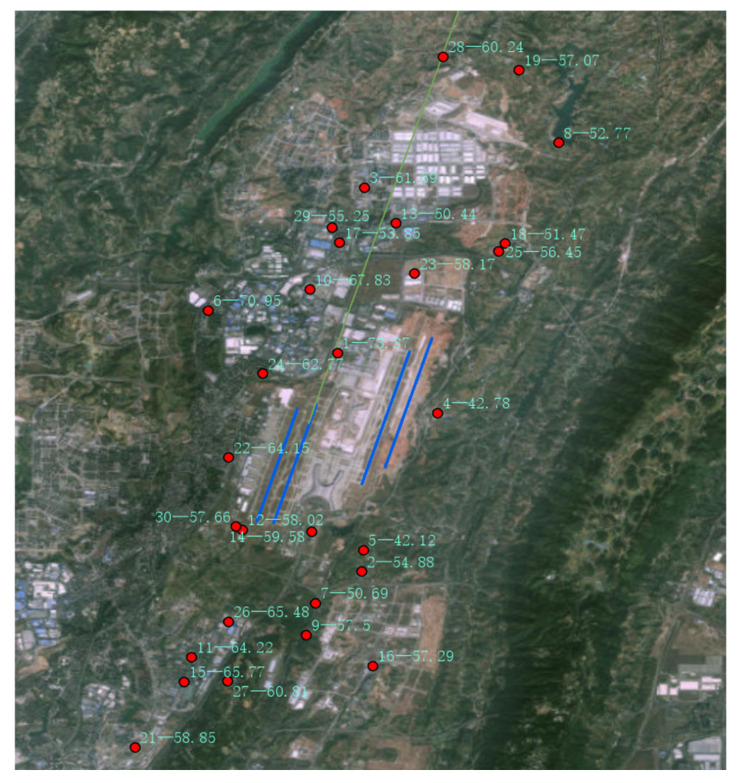
Map of noise spatial distribution.

For the noise distribution during this period, Figure 10 provides a corresponding visualization. In the figure, the red monitoring sites are marked with “-”, where the number before the “-” is the monitoring site number, and the number after the “-” is the noise value at that point. This marking method facilitates analysis by referring to the Label field values in Table 5.

#### 4.4.2. Spatial Interpolation

The theoretical foundations of the Inverse Distance Weighting (IDW) and Kriging interpolation methods were elaborated in Section 2.3. Based on the equivalent noise values from the monitoring stations listed in Table 5, this section will conduct interpolation calculations to construct a spatial distribution model. The spatial scope is defined by the impact zones at both ends along the runway.

- Inverse Distance Weighting (IDW)

**Figure 11:**
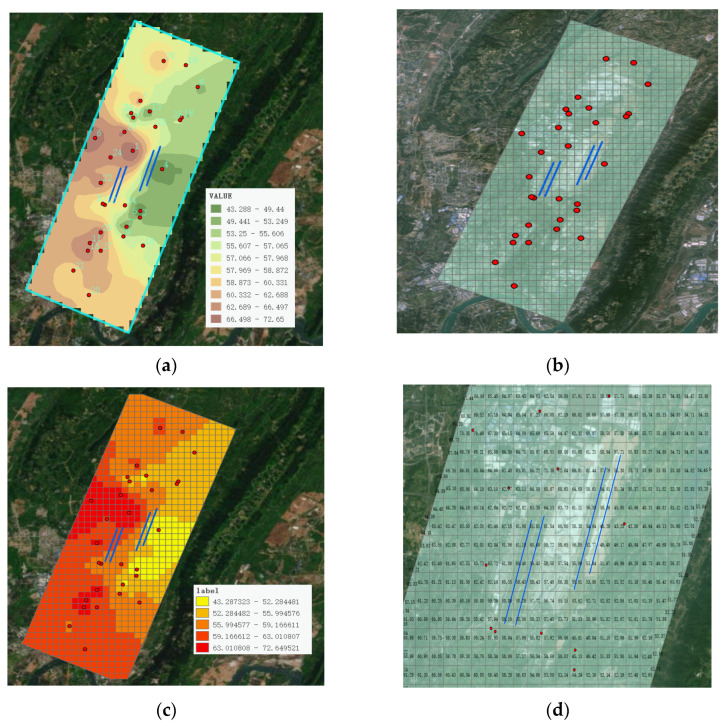
IDW spatial interpolation: (**a**) Raster map after IDW interpolation; (**b**) The surface raster noise values are mapped to the grid; (**c**) Zoomed-in grid view; (**d**) Color rendering of grid noise levels in segments.

This study performed IDW interpolation on the noise monitoring values of the monitoring stations listed in Table 5, with the results shown in Figure 11. The red dots represent the noise monitoring sites, while the blue lines denote the airport runway.

The specific operational steps are as follows:

Firstly, a dataset of the thirty noise monitoring sites was constructed, and the power function parameter was set to two. Subsequently, for the search domain selection, the standard tool was used (other types such as smooth, standard circular, and smooth circular can also be selected); the maximum number of neighboring elements was set to 15, the minimum number of neighboring elements was set to 10, the sector type was selected as single sector (four sectors or eight sectors can also be chosen), and the angle parameter was set to zero. Based on the above parameters, the IDW interpolation was completed, and the surface raster was outputted, as shown in Figure 11a.

For the output surface raster, the interpolation effect can be observed, where the noise values are evenly distributed across the entire surface raster plane through the interpolation process. To more intuitively display the noise data, this study further constructed a grid for the entire range and mapped the noise values from the interpolated surface raster into this grid, as shown in Figure 11b. By zooming in on the grid, the distribution of the interpolated noise values can be clearly observed, as shown in Figure 11c. In addition, this study segmented the grid based on the magnitude of noise values and rendered it with colors to distinguish different noise levels, as shown in Figure 11d.

- Kriging Interpolation

**Figure 12:**
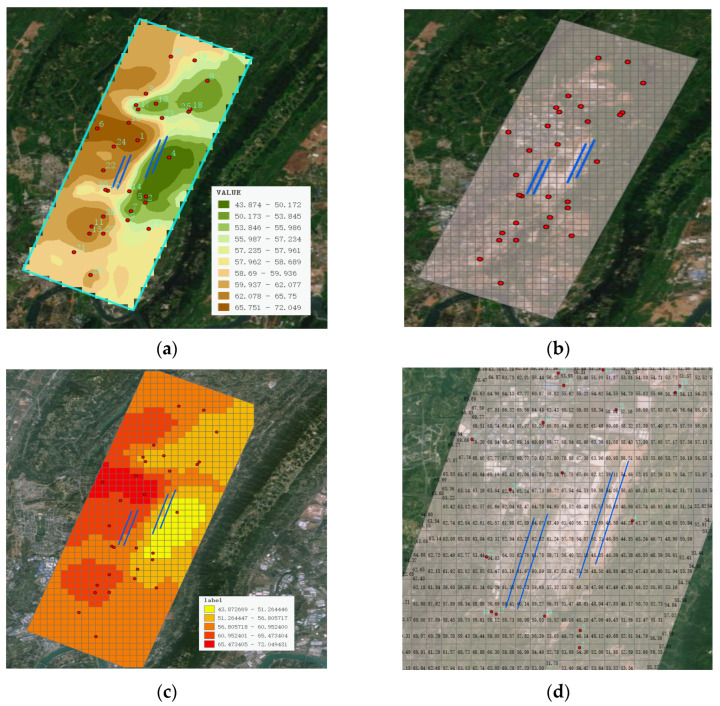
Kriging spatial interpolation: (**a**) Raster map after Kriging interpolation; (**b**) The surface raster noise values are mapped to the grid; (**c**) Zoomed-in grid view; (**d**) Color rendering of grid noise levels in segments.

Using a similar approach, this study performed Kriging interpolation analysis on the noise monitoring data of the monitoring stations listed in Table 5, with the results shown in Figure 12. The red dots represent the noise monitoring sites, while the blue lines denote the airport runway.

The specific operational steps include: Firstly, a dataset of the thirty noise monitoring sites was constructed; secondly, the spherical function was selected as the semivariogram model; then, the search radius was set, and 12 points were selected within the search range based on the variable; finally, the surface raster data were generated, as shown in Figure 12a.

Similar to the Inverse Distance Weighting (IDW) interpolation method, this study further constructed a grid model and assigned noise values obtained through interpolation calculations to each grid cell. Subsequently, the grid was segmented and rendered based on the magnitude of the noise values to achieve an intuitive visual effect. The specific rendering effects are shown in Figure 12b–d.

- Results Analysis

Specifically, Inverse Distance Weighting (IDW) interpolation has been performed for the noise values of the 30 noise monitoring sites, with the theoretical mathematical model presented in Equations (1) and (2). Kriging Interpolation has also been applied, and its theoretical mathematical model was detailed in Equations (3)–(6). The interpolation processes and results were then meticulously executed in GIS.

According to interpolation theory, the Inverse Distance Weighting (IDW) interpolation method is based on the “proximity similarity” principle, which means that points closer to the interpolation point have a greater influence on it. The weights are proportional to the inverse of the distances. Kriging interpolation is a geostatistical interpolation method that uses the spatial autocorrelation of regionalized variables for interpolation. By constructing a variogram model, it provides a linear unbiased and optimal estimation for unknown points. Despite the widespread application of IDW and Kriging interpolation methods across various fields, their application in airport noise monitoring remains highly novel and presents unique challenges. Airport noise monitoring requires the consideration of numerous complex factors, including meteorological conditions, flight dynamics, and topographical features. These factors necessitate targeted optimization and adjustment of traditional interpolation methods to ensure their effectiveness in this specific context.

Overall, Kriging interpolation, which quantifies spatial autocorrelation through the variogram, can more accurately reflect the spatial distribution patterns of noise and performs well when dealing with complex noise propagation patterns (e.g., noise distribution affected by meteorological conditions). However, it has a higher computational complexity, especially when the number of monitoring points is large, leading to a significant increase in computation time. It is also more reliant on the variogram model, and improper model selection may reduce interpolation accuracy.

In contrast, Inverse Distance Weighting (IDW) interpolation has a relatively simpler computational process, is easy to implement, and is suitable for handling large-scale datasets, especially when the sample points are relatively evenly distributed. However, this method calculates weights based solely on distance and does not take into account the statistical properties of spatial data, which may lead to less accurate interpolation results. It is also more sensitive to outliers, where the presence of outliers at known points can directly affect the interpolation results.

From the noise interpolation distribution maps, it can be observed that the noise distribution maps generated by Kriging interpolation are smooth and continuous, clearly revealing the spatial variation trends of noise. In areas with sparse monitoring points, Kriging interpolation optimizes the prediction results through the variogram model, avoiding over-reliance on local data.

The noise distribution maps generated by IDW interpolation exhibit higher accuracy near known points. However, in areas with sparse monitoring points, the “bull’s-eye effect” may occur. This effect is characterized by the interpolation results forming concentric circles around known points. The interpolation results are highly dependent on known points and do not fully reflect the spatial autocorrelation of noise.

In interpolation calculations, both Kriging and IDW possess their respective strengths. However, IDW generally exhibits larger errors, particularly in areas with sparse monitoring points, where the interpolation results tend to be less stable. Conversely, Kriging outperforms IDW in terms of accuracy and reliability, offering a more precise depiction of the spatial distribution patterns of noise, especially in regions with sparse monitoring points.

In practical applications, the choice of interpolation method should be based on specific requirements. For instance, in airport noise monitoring, Kriging interpolation is recommended for generating high-precision noise distribution maps to support noise management and policy-making, whereas in real-time data processing, IDW can be combined to improve computational efficiency.

## 5. Discussion on Error Assessment and Model Optimization

### 5.1. Error Origins

In this study, several potential sources of error may impact the accuracy and reliability of the airport noise monitoring system:

- Measurement Error from Sensors

The precision of noise sensors, weather sensors, and ADS–B receivers is crucial for the reliability of the collected data. Despite the high quality of the sensors employed in this study, intrinsic measurement errors remain inevitable. Specifically, both noise sensors and weather sensors are subject to certain degrees of measurement error. These errors are initially introduced during the data acquisition phase and can subsequently propagate through the analysis processes.

- Error in Data Fusion

The integration of noise data, weather data, and ADS–B data necessitates timestamp alignment and data synchronization. However, the varying update frequencies and transmission delays of different sensors can introduce discrepancies in timestamp matching, which may subsequently compromise the accuracy of correlating noise events with flight information.

- Error in Spatial Interpolation

The Kriging and Inverse Distance Weighting (IDW) methods, both employed for spatial interpolation, are prone to introducing errors. IDW exhibits heightened sensitivity to outliers, which may result in a “bull’s-eye effect” in regions characterized by sparse monitoring points. While Kriging demonstrates superior performance in capturing spatial autocorrelation, it is associated with greater computational complexity and a higher dependency on the selection of the variogram model. An improper choice of model may consequently lead to a reduction in interpolation accuracy.

### 5.2. Methodology for Error Assessment

To assess the accuracy of the spatial interpolation models and the overall monitoring system, the following methods can be employed:

- Cross-Validation

The performance of the Kriging and IDW interpolation methods was assessed via cross-validation. Specifically, the dataset was partitioned into a training set and a validation set. The interpolation models were subsequently trained using the training set. Thereafter, the predicted values generated by these models were compared with the actual values in the validation set to evaluate their accuracy and reliability.

- Comparison with Empirical Data

The interpolated noise distribution map was juxtaposed against the actual noise measurements obtained from established monitoring sites. This direct comparison served to evaluate the model’s capacity to accurately predict noise levels in regions devoid of direct measurements.

- Sensitivity Analysis

A sensitivity analysis was performed to assess the influence of various variogram models (e.g., spherical, exponential, and Gaussian) on the outcomes of Kriging interpolation. This analysis facilitates the identification of the most appropriate variogram model for the dataset and ensures the robustness of the interpolation model to variations in model parameters.

### 5.3. Strategies for Model Optimization

To improve the accuracy and reliability of the airport noise monitoring system, the following optimization strategies can be employed:

- Optimization of Sensor Calibration and Layout

Regular calibration of sensors is crucial for maintaining their measurement accuracy. Furthermore, optimizing the layout of the monitoring stations to achieve a more uniform distribution of sensors across the study area can mitigate bias in spatial interpolation and improve the overall accuracy of the noise distribution maps.

- Data Preprocessing and Quality Control

The raw data underwent rigorous preprocessing, which encompassed noise filtering, imputation of missing values, and standardization of data formats. Additionally, a time-synchronization algorithm was utilized to ensure the precise alignment of data from disparate sources, thereby mitigating errors in data fusion.

- Hybrid Interpolation Model

A hybrid approach that integrates Kriging and IDW was investigated to capitalize on the strengths of each method. IDW was employed in regions with dense monitoring points, capitalizing on its computational efficiency. Conversely, Kriging was implemented in sparsely monitored areas to more effectively capture spatial autocorrelation. This hybrid model successfully balanced accuracy and computational efficiency.

- Integration of Machine Learning

Future research could explore the integration of machine learning algorithms, such as random forests and gradient boosting, into the spatial interpolation process. These algorithms could be trained using historical noise data to predict noise levels at unmonitored locations. Machine learning models have the potential to capture the complex nonlinear relationships between noise levels and influencing factors, such as meteorological conditions and flight paths, thereby enhancing the accuracy of noise distribution maps.

- Three-dimensional noise modeling

Initial efforts have been made to develop a three-dimensional noise modeling framework, which takes into consideration the vertical propagation characteristics of noise. By incorporating topographical elevation data and building heights, this approach provides a more comprehensive depiction of noise distribution across both horizontal and vertical dimensions.

## 6. Conclusions

With the acceleration of global urbanization, airport noise pollution has emerged as a salient environmental issue, exerting profound impacts on residents’ health and the ecological environment. Traditional airport noise monitoring systems are characterized by significant limitations in spatial coverage, real-time data acquisition, data correlation, and cost-effectiveness, thereby impeding their ability to meet the current demands for refined monitoring and management of airport noise.

This study systematically constructed an airport noise monitoring framework from multiple dimensions, including multi-source data integration, Internet of Things (IoT) sensing technology, noise event correlation analysis, and spatial interpolation methods. By integrating multi-source sensor data such as noise, weather, and ADS–B, and establishing correlations between noise data and meteorological and flight data, this study achieved deep integration of multi-source data and precise tracing of noise events. Kriging and Inverse Distance Weighting (IDW) methods were employed for spatial interpolation of sparse monitoring point data to generate high-resolution noise distribution maps and establish a spatial distribution model of airport noise. The results demonstrate that the proposed methods can achieve refined monitoring, precise tracing, and management of noise, thereby improving the utilization efficiency of noise monitoring data. This approach not only enables a more comprehensive analysis of the spatiotemporal distribution characteristics of airport noise but also provides technical support for the harmonious development between airports and surrounding communities.

The research findings have been implemented at Chongqing Jiangbei International Airport, where the deployment of 30 monitoring stations has validated the reliability of the system. The results reveal that the multi-source data fusion technology and spatial distribution modeling methods can accurately reflect the spatiotemporal distribution patterns of airport noise. This study demonstrates significant advantages in the identification, tracing, and spatial distribution prediction of noise events, providing robust data support for the formulation of airport noise management policies. The multi-source data fusion framework introduced in this study exhibits broad applicability, extending beyond specific airports to other environmental monitoring contexts, including urban traffic noise and industrial noise. By consolidating a variety of data sources, this framework enables a more holistic analysis of noise spatiotemporal distribution and correlation characteristics. This not only provides a novel technological approach for environmental noise monitoring but also offers valuable references for similar application scenarios. Through the sharing of data and experiences, different airports can learn from one another and collaboratively improve their noise monitoring and management capabilities. The high-resolution noise distribution maps and noise event attribution analyses generated by this study can serve as robust scientific evidence for the development of airport noise management policies.

Constrained by topographical conditions and equipment costs, the layout of monitoring sites may compromise the global accuracy of interpolation models. Future research endeavors should focus on optimizing the placement of these monitoring sites. By leveraging Geographic Information Systems (GIS) and topographical analysis, the strategic planning of monitoring site locations can ensure the representativeness and comprehensiveness of the collected data. Additionally, the nonlinear relationship between meteorological conditions and noise propagation warrants further modeling and optimization. Future studies could benefit from incorporating machine learning algorithms, such as spatial random forests, to enhance the generalization ability of interpolation models. These algorithms can automatically identify and process complex patterns in noise data, thereby improving model accuracy and robustness. Furthermore, exploring three-dimensional noise distribution modeling to support vertical spatial analysis could provide valuable insights into the propagation patterns of noise at different altitudes.

In summary, this study has realized specific technical innovations and confirmed their effectiveness through practical applications. By amalgamating multi-source data fusion with spatial distribution modeling, a pioneering method for airport noise monitoring has been established. These accomplishments are not confined to particular airports; they are scalable, facilitating their application to other airports and offering a benchmark for other environmental monitoring domains. It is anticipated that subsequent research and application will offer further reinforcement for the evolution of airport noise management and environmental noise monitoring technologies.

## Figures and Tables

**Table 1:** Hourly monitoring data for Noise Monitoring Site 1 on 9 January 2025.

Time (h)	Leq (dBA)	Lmax (dBA)	Lmin (dBA)	L10 (dBA)	L50 (dBA)	L90 (dBA)
00:00:00	57.21895833	82.4	44.2	54.24346	50.136524	48.185143
01:00:00	67.82280027	96.2	44	52.802624	50.22635	47.000862
02:00:00	64.63856114	89.7	45.5	55.840534	53.27159	48.806835
03:00:00	60.8378527	87.8	43.6	53.680344	51.814022	47.4408
04:00:00	51.38262315	64.7	43.9	53.04144	51.911835	47.67696
05:00:00	54.37033719	76.1	45.5	54.31284	52.329857	48.197487
06:00:00	66.77060558	83.7	45.2	72.953186	54.26121	48.164143
07:00:00	69.73739269	88.7	48	73.70467	65.7477	53.58906
08:00:00	69.28191455	86.6	51.6	74.84679	61.018543	55.889618
09:00:00	68.9503474	85.7	52.6	74.0052	60.647236	55.952194
10:00:00	69.40169101	86.7	52.8	74.13598	55.73404	54.491386
11:00:00	68.28523386	86.8	51.4	74.267624	57.30418	54.43346
12:00:00	68.8080414	87.7	53.2	74.33332	57.060947	55.21252
13:00:00	71.56009479	102.3	52.8	75.558815	59.49364	55.667023
14:00:00	69.07133718	89.2	52.9	74.81701	58.9122	55.083817
15:00:00	66.97292174	84	50.2	72.708466	56.203506	54.206123
16:00:00	67.45932165	85.2	52.1	73.15712	55.963203	54.298683
17:00:00	69.05715316	86.1	49.6	75.04843	54.95877	52.631676
18:00:00	66.28781213	85.4	49.2	73.38589	52.93705	51.523018
19:00:00	67.47925763	84.6	48.6	74.03604	53.514595	51.131668
20:00:00	69.17686812	88.2	48.3	73.82557	56.62759	51.511982
21:00:00	65.50019676	86.5	49	72.037254	52.73172	50.955578
22:00:00	67.14169909	85.7	48.5	73.864075	54.404133	50.53803
23:00:00	64.50416703	83.3	48.8	70.41695	54.894753	51.10674

**Table 2:** All-Day meteorological monitoring data on 9 January 2025.

Time (hh)	Wind Speed (m/s)	Wind Direction (°)	Temperature (°C)	Atmospheric Pressure (Pa)	Relative Humidity (%)	Precipitation (mm)
00:00:00	0.001	7.003	7.342	984.522	84.635	14.576
01:00:00	0.025	7.001	7.264	985.808	85.826	14.592
02:00:00	0.118	7.002	6.967	985.515	88.246	14.662
03:00:00	0.037	7	6.755	985.488	90.286	15.116
04:00:00	0.033	7.003	6.725	985.682	90.068	15.183
05:00:00	0.083	7	6.728	986.06	89.068	15.191
06:00:00	0.036	7.001	6.678	986.798	90.258	15.187
07:00:00	0.066	7.003	6.6	986.342	91.036	15.161
08:00:00	0.026	7.003	6.797	986.994	90.486	15.153
09:00:00	0.036	7.001	7.247	987.763	87.978	15.174
10:00:00	0.163	7.001	7.727	989.76	84.281	15.187
11:00:00	0.34	7.001	8.001	988.483	82.359	15.165
12:00:00	0.209	7.003	7.769	987.731	81.266	15.156
13:00:00	0.202	7.001	6.96	987.765	89.064	15.172
14:00:00	0.099	7.003	6.961	987.856	89.28	15.261
15:00:00	0.114	7.004	7.019	986.643	89.513	15.283
16:00:00	0.07	7.001	7.259	987.737	87.758	15.283
17:00:00	0.183	7.002	7.376	987.473	86.439	15.261
18:00:00	0.151	7.001	7.301	989.534	86.049	15.296
19:00:00	0.167	7.005	7.396	988.968	84.353	15.278
20:00:00	0.184	7.003	7.48	990.685	83.198	15.27
21:00:00	0.091	7.003	7.526	991.287	82.269	15.287
22:00:00	0.187	7.002	7.396	991.712	83.434	15.278
23:00:00	0.396	7	6.988	991.204	87.049	15.278

**Table 3:** Noise data from thirty noise monitoring sites at 12:00 on 9 January 2025.

Site Number	Leq (dBA)	Lmax (dBA)	Lmin (dBA)	L10 (dBA)	L50 (dBA)	L90 (dBA)
1	68.8080414	87.7	53.2	74.33332	57.060947	55.21252
2	59.06552721	76.2	38.8	65.3053	51.742054	46.71864
3	61.38223383	75.4	49.5	64.02256	60.247715	57.564903
4	45.85823244	65.1	36.7	48.717724	44.883797	41.740036
5	58.05757312	76.4	32	65.614296	42.77845	40.01837
6	67.18098721	83.4	53.2	72.28866	58.72051	55.11236
7	57.53974885	72.3	42.6	62.449257	53.430676	51.55485
8	57.53863789	75.9	47.3	61.515705	52.830418	50.91665
9	61.16363204	73.9	42.7	64.64619	59.766407	57.403732
10	64.09911025	76.7	55.5	67.68079	62.800972	58.178623
11	61.87966775	76.4	55.4	65.371315	60.16599	57.991085
12	60.54930981	77.2	50.8	64.80918	57.86744	54.276775
13	61.7346707	76.9	45.6	66.09535	55.975376	48.603966
14	60.67857799	70.2	56.6	62.006264	60.34982	59.245968
15	60.88050682	76.8	52.5	65.769424	56.69144	55.37898
16	58.06431252	71.5	49.3	59.89837	57.643604	55.127884
17	59.54150133	75.1	47.4	63.675392	55.168346	52.11068
18	59.90850949	76.2	15.4	65.106865	56.15736	51.361378
19	54.22695959	79.7	26.8	57.09515	49.00404	40.911175
20	59.99734105	77	53.9	61.487232	59.84029	58.0622
21	57.81065247	69.2	54.2	59.731377	56.828323	56.047817
22	65.53735521	76.1	60.9	66.87146	65.30715	64.152336
23	63.86458017	82.8	46.2	66.70404	62.073383	55.175987
24	62.76494539	78.9	54.3	64.296875	62.417725	60.666344
25	58.84833311	75.1	47.9	63.01381	56.4168	54.608795
26	61.21560683	80.6	45.7	67.029175	53.304443	49.730385
27	58.282585	75.5	54.8	60.78647	57.0738	56.04345
28	56.25931136	76.9	25.2	60.242733	44.313698	31.132519
29	59.02602687	75	46.1	63.767994	55.144184	49.134953
30	59.10325463	76.3	50	62.489525	57.380215	54.55352

**Table 4:** Noise event table for Noise Monitoring Site 1.

No.	Incident Type	Start Time (hh:mm:ss)	Peak Time (hh:mm:ss)	Duration (s)	Leq (dBA)	SEL (dBA)	Lmax (dBA)	Connecting Flights
1	Aviation incident	12:06:05	12:06:25	45	75.36	86.50	83.60	9C6196
2	Aviation incident	12:07:40	12:08:05	50	76.62	88.08	85.10	MU2926
3	Aviation incident	12:09:27	12:09:47	41	72.07	83.83	79.20	CZ3466, MF8472
4	Aviation incident	12:13:25	12:13:47	46	75.15	86.91	83.30	DZ6285
5	Aviation incident	12:15:14	12:15:36	49	74.37	86.41	82.00	3U3209
6	Aviation incident	12:18:05	12:18:24	46	76.02	86.81	84.40	CA1432
7	Aviation incident	12:20:10	12:20:25	41	72.28	82.69	80.70	8L9606
8	Non-aviation incident	12:21:37	12:21:55	46	75.55	85.55	84.10	--
9	Aviation incident	12:23:13	12:23:30	45	74.00	85.46	81.30	PN6317
10	Non-aviation incident	12:28:14	12:28:34	44	75.48	86.94	82.70	--

**Table 5:** Monitoring data for all monitoring sites at 12:15 on 9 January 2025.

Site Number	Data_Time (hh:mm:ss)	Noise_Value (dBA)	Label
1	2025-01-09 12:15	73.37	1—73.37
2	2025-01-09 12:15	54.88	2—54.88
3	2025-01-09 12:15	61.59	3—61.59
4	2025-01-09 12:15	42.78	4—42.78
5	2025-01-09 12:15	42.12	5—42.12
6	2025-01-09 12:15	70.95	6—70.95
7	2025-01-09 12:15	50.69	7—50.69
8	2025-01-09 12:15	52.77	8—52.77
9	2025-01-09 12:15	57.5	9—57.5
10	2025-01-09 12:15	67.83	10—67.83
11	2025-01-09 12:15	64.22	11—64.22
12	2025-01-09 12:15	58.02	12—58.02
13	2025-01-09 12:15	50.44	13—50.44
14	2025-01-09 12:15	59.58	14—59.58
15	2025-01-09 12:15	65.77	15—65.77
16	2025-01-09 12:15	57.29	16—57.29
17	2025-01-09 12:15	53.85	17—53.85
18	2025-01-09 12:15	51.47	18—51.47
19	2025-01-09 12:15	57.07	19—57.07
20	2025-01-09 12:15	60.25	20—60.25
21	2025-01-09 12:15	58.85	21—58.85
22	2025-01-09 12:15	64.15	22—64.15
23	2025-01-09 12:15	58.17	23—58.17
24	2025-01-09 12:15	62.77	24—62.77
25	2025-01-09 12:15	56.45	25—56.45
26	2025-01-09 12:15	65.48	26—65.48
27	2025-01-09 12:15	60.81	27—60.81
28	2025-01-09 12:15	60.24	28—60.24
29	2025-01-09 12:15	55.25	29—55.25
30	2025-01-09 12:15	57.66	30—57.66

## Data Availability

The original contributions presented in this study are included in this article; further inquiries can be directed to the corresponding author.
